# Fried food consumption and the risk of pancreatic cancer: A large prospective multicenter study

**DOI:** 10.3389/fnut.2022.889303

**Published:** 2022-07-22

**Authors:** Guo-Chao Zhong, Qian Zhu, Jian-Ping Gong, Dong Cai, Jie-Jun Hu, Xin Dai, Jun-Hua Gong

**Affiliations:** ^1^Department of Hepatobiliary Surgery, The Second Affiliated Hospital of Chongqing Medical University, Chongqing, China; ^2^Department of Epidemiology and Biostatistics, School of Public Health and Management, Chongqing Medical University, Chongqing, China

**Keywords:** fried foods, chips, pancreatic cancer, prospective study, nutritional epidemiology

## Abstract

**Background and aims:**

Whether fried food consumption is associated with the risk of pancreatic cancer remains elusive. We aimed to examine this association in a US population.

**Methods:**

A population-based cohort of 101,729 US adults was identified. Fried food consumption was assessed with a validated food frequency questionnaire. Hazard ratios (HRs) with 95% confidence intervals (CIs) were calculated. Explanatory analyses were conducted to identify main contributor(s) to the observed association.

**Results:**

During an average follow-up of 8.86 years (900871.2 person-years), 402 pancreatic cancer cases occurred. High consumption of total fried foods (deep-fried plus pan-fried foods; HR_quartile4 vs. 1_ 0.71, 95% CI 0.51–0.99, *P*_trend_ = 0.047) and deep-fried foods (HR_quartile 4 vs. 1_ 0.64, 95% CI 0.47–0.88, *P*_trend_ = 0.011), but not pan-fried foods (HR_quartile 4 vs. 1_ 0.98, 95% CI 0.73–1.32; *P*_trend_ = 0.815), was found to be associated with a reduced risk of pancreatic cancer in a non-linear dose–response manner, which was not modified by predefined stratification factors and persisted in sensitivity analyses. In explanatory analyses, only chip consumption was found to be inversely associated with the risk of pancreatic cancer; consistently, the initial significant associations between total fried food and deep-fried food consumption and the risk of pancreatic cancer changed to be non-significant after omitting or further adjusting for chip consumption.

**Conclusion:**

Consumption of deep-fried foods, but not pan-fried foods, is inversely associated with the risk of pancreatic cancer in this US population. The role of deep-fried foods in reducing the risk of pancreatic cancer appears to be mainly attributable to chips. More studies are needed to confirm our findings in other populations and settings.

## Introduction

Frying is a commonly used cooking method in Western countries and makes foods more palatable and crunchy. Frying changes the composition of foods and their frying media through polymerization, hydrogenation, and oxidation (1), which produce some compounds thought to be potentially carcinogenic (e.g., acrylamide and heterocyclic amines). The potential carcinogenic role of fried foods has been suggested by several case-control studies, all of which revealed a positive association between fried food consumption and the risk of cancer, including pharyngolaryngeal cancer (2, 3), gastric cancer (4), gallbladder cancer (5), and prostate cancer (6). Moreover, individuals with high fried food consumption were found to be at elevated risks of obesity and type 2 diabetes (7, 8), two well-known risk factors for cancer (9). Together, these data suggest that fried foods are potentially carcinogenic.

Pancreatic cancer is the third most common cause of cancer-related mortality in the US, with an estimated 48,220 cancer deaths in 2021 (10). Diets have been indicated to play an important role in the etiology of this cancer (11), which is in accordance with our recent findings on dietary behaviors and pancreatic cancer (12–14). The association of fried food consumption with the risk of pancreatic cancer has been evaluated in an early case-control study in Canada, with a positive association observed (15). However, this study has a small sample size (418 individuals), which makes it be prone to small study effects (i.e., small studies tend to show larger risk estimates and are performed with less methodological rigor than large studies) (16). More importantly, case–control studies are susceptible to recall bias and cannot establish a temporal association.

Clarifying the potential association between fried food consumption and the risk of pancreatic cancer is critical for public health, considering the dismal prognosis of this cancer and that more than one-third of Americans patronize fast food restaurants daily where fried foods comprise the majority of items sold (8, 17). Therefore, we performed a prospective multicenter cohort study to test an *a priori* hypothesis that high fried food consumption is associated with an increased risk of pancreatic cancer in a US population.

## Materials and methods

Our results were reported following the Strengthening the Reporting of Observational Studies in Epidemiology statement (18).

### Study population

**FIGURE 1:**
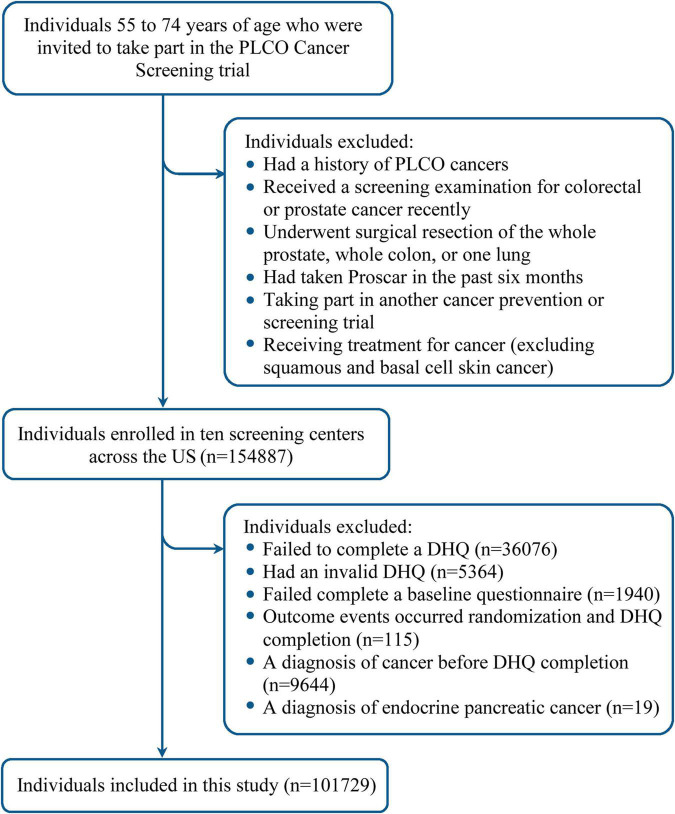
Flow chart identifying participants included in this study. PLCO, Prostate, Lung, Colorectal, and Ovarian; DHQ, diet history questionnaire.

The study population was derived from the Prostate, Lung, Colorectal, and Ovarian (PLCO) Cancer Screening Trial, which was developed for determining the potential beneficial roles of selected screening tests in reducing mortality from PLCO cancers. The PLCO Cancer Screening Trial is a multicenter randomized clinical study, whose study design has been described elsewhere (19). Briefly, between November 1993 and September 2001, individuals 55–74 years of age were invited to participate in this trial in ten screening centers across the US. As shown in Supplementary Table 1, there were significant differences in some sociodemographic characteristics (e.g., sex and ethic group) between study centers, indicating that study population of the PLCO Cancer Screening Trial was heterogeneous at the level of study center. A total of 154,887 individuals were finally enrolled based on the predefined eligibility criteria (Figure 1). Enrolled individuals were individually randomized to the intervention group or the control group in equal proportions, with those in the intervention group receiving screening tests for PLCO cancers while those in the control group receiving usual care. The PLCO Cancer Screening Trial was approved by institutional review boards of the US National Cancer Institute and each screening center. All participants provided written informed consent.

In this study, following participants were further excluded: (1) 36,076 participants failing to complete a diet history questionnaire (DHQ); (2) 5,364 participants with an invalid DHQ, which refers to death date prior to DHQ completion date, more than eight missing frequency responses, missing the date of completion, and/or extreme values of energy intake (the first or last percentile); (3) 1,940 participants failing to complete a baseline questionnaire; (4) 115 participants with outcome events (incident pancreatic cancer, loss to follow-up, or death) observed between randomization and DHQ completion; (5) 9,644 participants receiving a diagnosis of cancer before DHQ completion; and (6) 19 participants with a diagnosis of endocrine pancreatic cancer. Finally, a total of 101,729 participants were qualified for inclusion (Figure 1). Notably, a comparison between included and excluded populations found that standardized differences of most sociodemographic characteristics were less than 0.1 (20), indicating that the potential for non-participation bias resulting from the exclusion of a huge number of participants was low (Supplementary Table 2).

### Dietary assessment

Dietary assessment was performed at baseline using the aforementioned DHQ (version 1.0, National Cancer Institute), which is a 124-item self-reported food frequency questionnaire used for evaluating the frequency and portion size of an individual’s food consumption during the past year. The Eating at America’s Table Study had compared the performance of the DHQ with four 24-h dietary recalls in a nationally representative sample of 1,640 subjects; the corresponding results showed that the DHQ had good performance in the evaluation of dietary intake (21). Daily food consumption was approximated by multiplying food frequency by portion size; daily intakes of nutrients and energy were estimated on the basis of two nutrient databases, namely Nutrition Data Systems for Research (22) and US Department of Agriculture’s 1994–1996 Continuing Survey of Food Intakes by Individuals (23). Healthy Eating Index-2015, a frequently used index reflecting an individual’s diet quality, was computed as described previously (24). To eliminate the extraneous variation of dietary data caused by energy intake, food consumption and nutrient intake were adjusted for energy intake using the residual method before the formal statistical analyses (25).

Fried foods in the DHQ were categorized into deep-fried and pan-fried foods. A given fried food was assumed to be deep-fried if its frying type was not mentioned (26). Thus, in this study, deep-fried chicken, fried fish (including fried seafood or shellfish), fried potato, and chips were classified into deep-fried foods, while pan-fried bacon, pan-fried chicken, pan-fried hamburger, pan-fried pork chops, pan-fried sausage, and pan-fried steak were classified into pan-fried foods. Here, fried potatoes referred to French fries, home fries, tater tots, or hash browns; chips referred to potato chips, tortilla chips, or corn chips. To investigate the potential impacts of doneness degree, we further divided pan-fried foods into the following three categories: just done, well done, and very well done. Total fried food consumption was calculated as the sum of consumption of deep-fried and pan-fried foods.

### Ascertainment of pancreatic cancer

Pancreatic cancer was ascertained predominantly through an annual study update form, which was mailed to participants by each screening center for inquiring whether they were diagnosed with pancreatic cancer, and if so, the date and location of diagnosis and contact information of their healthcare providers. Cancers reported on the annual study update form were further ascertained by scrutinizing any available medical records. In addition, death certificates and family reports were used as Supplementary Material for cancer ascertainment. To reduce the heterogeneity of pancreatic cancer cases, only participants with a diagnosis of exocrine pancreas cancer (ICD-O-2 codes: C25.0-C25.3 and C25.7-C25.9) were considered.

### Assessment of non-dietary variables

Sex, ethnicity, educational degree, body weight, height, smoking status, pack-years, history of diabetes, family history of pancreatic cancer, and aspirin use, were assessed with a self-reported baseline questionnaire. Body mass index was computed by dividing body weight (kg) by height squared (m2). Physical activity level was expressed as total time of moderate-to-vigorous activity each week, which was assessed through a self-reported supplemental questionnaire. Age at DHQ completion and alcohol consumption were assessed with the above DHQ.

### Statistical analysis

To reduce the potential biases and increase the statistical power, we used the following methods to impute missing values. Specifically, for variables with less than 5% missing values, we used the modal value and the median to impute missing values of categorical and continuous variables, respectively; for the variable “physical activity level” that had 25.69% missing values, we assumed that these values were missing at random and then used multiple imputation with chained equations to impute them, with the number of imputations set at 25 (27). The distribution of variables with missing values before and after imputation is shown in Supplementary Table 3. To examine the potential influence of data imputation on our results, main statistical analyses were repeated in the population with complete covariate data.

**FIGURE 2:**
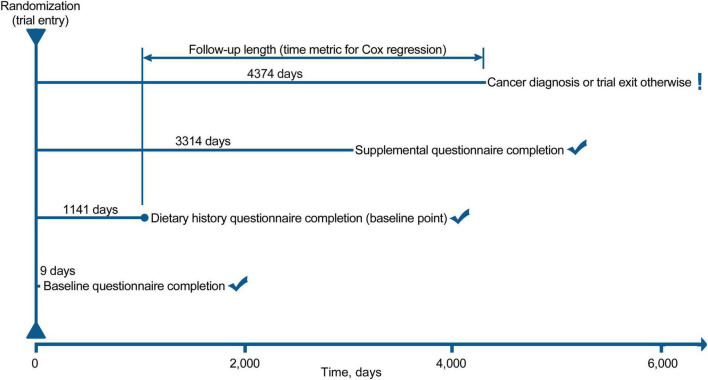
The timeline and follow-up scheme of our study.

Cox proportional hazards regression was used to compute hazard ratios (HRs) with 95% confidence intervals (CIs) of the association of fried food consumption with the risk of pancreatic cancer, with follow-up length as time metric. Follow-up length was computed from the date of DHQ completion to the date of pancreatic cancer diagnosis, study dropout, death, or the end of follow-up (December 31, 2009), whichever occurred first (Figure 2). In regression analyses, fried food consumption was split into quartiles and the first quartile was set as the reference group. A P for linear trend across quartiles was computed by modeling the median of fried food consumption in each quartile as a continuous variable. The proportional hazards assumption of Cox regression was found to be satisfied after examination of Schoenfeld residuals (*P* for global test > 0.05) (28). As recommended, covariate selection for multivariable analyses was based on our causal knowledge of the existing literature rather than statistical criteria (29). Specifically, model 1 adjusted for age and sex; model 2 further adjusted for well-known factors associated with the risk of pancreatic cancer, namely smoking status, alcohol consumption, body mass index, aspirin use, history of diabetes, family history of pancreatic cancer, and energy intake from diet. To reflect how robust the observed association was to the unmeasured confounding, the *E*-value was computed using an online calculator^1^ (30). The *E*-value estimates what the minimum HR would have to be for any unmeasured confounder to negate the observed association of fried food consumption with the risk of pancreatic cancer (30).

Restricted cubic spline regression with knots located at the 10th, 50th, and 90th percentiles were used to explore the potential dose–response association between fried food consumption and the risk of pancreatic cancer, with the reference level set at 0 g/day. Of note, the choice of the number of knots was based on the Akaike’s information criterion in this study (31), with the lowest value indicating the best fitted model. To eliminate the potential impacts of extreme values, participants with extreme fried food consumption (top 2.5% or bottom 2.5%) were excluded from the dose–response analysis. A *P* for non-linearity was computed by testing whether the estimated value of the second spline equals 0.

To determine the potential effect modifiers of the observed association of fried food consumption with the risk of pancreatic cancer, prespecified subgroup analyses were performed after stratifying for age (≥ 65 vs. < 65 years), sex (male vs. female), body mass index (≥ 25 vs. < 25), aspirin use (yes vs. no), smoking status (current or past vs. never), alcohol consumption (≥ median vs. < median), and trial group (intervention vs. control groups). A *P* for interaction was computed by comparing models with and without interaction terms prior to conducting the formal subgroup analyses to avert possibly spurious subgroup differences.

The following sensitivity analyses were performed to evaluate the robustness of our results: (1) excluded participants with extreme values of energy intake, defined as < 800 or > 4,000 kcal/day for males and < 500 or > 3,500 kcal/day for females (32); (2) excluded participants with extreme fried food consumption as defined as above; (3) excluded pancreatic cancer cases observed within the first 2 years of follow-up to test the potential influence of reverse causation; (4) excluded participants receiving a diagnosis of pancreatic cancer following other cancer diagnoses; (5) repeated the analysis with energy-unadjusted fried food consumption; (6) repeated the analysis with sex-specific quartiles, as the distribution of fried food consumption was found to be different by sex; (7) further adjusted for Healthy Eating Index-2015 on model 2 to test whether the observed association was mediated by diet quality; and (8) further adjusted for physical activity level and consumption of fruits, vegetables, red and processed meat, and coffee on model 2.

To identify the main contributor(s) to the observed association, the following explanatory analyses were performed: (1) examined the association of individual fried food consumption with the risk of pancreatic cancer; (2) examined the association of fried food consumption with the risk of pancreatic cancer after further adjusting for or omitting an individual fried food. A statistical significance level of *P* < 0.05 was used under a two-tailed test. All statistical analyses were performed using STATA software (version 12.0, StataCorp LP, College Station, Texas).

## Results

### Participant characteristics

The mean (standard deviation) energy-adjusted consumption of total fried foods, deep-fried foods, and pan-fried foods in the whole study population was 24.3 (28.7) g/day, 17.2 (23.2) g/day, and 7.1 (11.9) g/day, respectively. Baseline characteristics of study population are shown in Table 1. Compared with participants in the lowest quartile of total fried consumption, those in the highest quartile were more likely to be male, be current or past smokers, and be aspirin users, had lower levels of education and physical activity and lower Healthy Eating Index-2015 but had higher body mass index, alcohol consumption, and energy intake from diet, and were more likely to have a history of diabetes. Moreover, participants in the highest vs. the lowest quartiles of total fried food consumption had higher consumption of vegetables, red processed meat, coffee, and nuts but lower consumption of fruits. When study population was classified by deep-fried food or pan-fried food consumption, a similar phenomenon was observed (data not shown).

**TABLE 1:** Characteristics of study population based on quartiles of energy-adjusted total fried food consumption*^a^*.

Characteristics	Quartiles of energy-adjusted total fried food consumption, range (mean), g/day				
	<6.10 (2.6)	6.1 – <15.1 (10.2)	15.1 – ≤31.8 (22.2)	>31.8 (61.9)	*P* for differences across quartiles
Number of participants	25,433	25,432	25,432	25,432	
Age (years)	66.5 ± 5.9	65.8 ± 5.7	65.3 ± 5.6	64.4 ± 5.5	<0.001
Male	7,526 (29.6)	10,577 (41.6)	13,503 (53.1)	17,875 (70.3)	<0.001
**Ethnic group**					
Non-Hispanic white	23,111 (90.9)	23,253 (91.4)	23,211 (91.3)	22,937 (90.2)	<0.001
Non-Hispanic black	754 (3.0)	677 (2.7)	830 (3.3)	1,089 (4.3)	
Hispanic	364 (1.4)	351 (1.4)	352 (1.4)	428 (1.7)	
Others*^b^*	1,204 (4.7)	1,151 (4.5)	1,039 (4.1)	978 (3.8)	
**Educational degree**					
College below	14,643 (57.6)	15,995 (62.9)	16,624 (65.4)	17,673 (69.5)	<0.001
College graduate	4,945 (19.4)	4,590 (18.0)	4,357 (17.1)	3,950 (15.5)	
Postgraduate	5,845 (23.0)	4,847 (19.1)	4,451 (17.5)	3,809 (15.0)	
Body mass index*^c^*	26.0 ± 4.6	27.0 ± 4.7	27.6 ± 4.7	28.3 ± 4.8	<0.001
Physical activity (min/week)*^d^*	131.4 ± 127.3	119.3 ± 119.8	118.1 ± 120.8	119.4 ± 122.7	<0.001
**Smoking status**					
**Current**					<0.001
>60 Pack-years	322 (1.3)	525 (2.1)	847 (3.3)	1,210 (4.8)	
30–60 Pack-years	672 (2.6)	1,006 (4.0)	1,172 (4.6)	1,430 (5.6)	
<30 Pack-years	482 (1.9)	567 (2.2)	582 (2.3)	584 (2.3)	
**Past**					
>60 Pack-years	959 (3.8)	1,195 (4.7)	1,434 (5.6)	2,052 (8.1)	
30–60 Pack-years	2,522 (9.9)	2,838 (11.2)	3,135 (12.3)	3,617 (14.2)	
<30 Pack-years	6,747 (26.5)	6,464 (25.4)	6,514 (25.6)	6,283 (24.7)	
Never	13,729 (54.0)	12,837 (50.5)	11,748 (46.2)	10,256 (40.3)	
Alcohol consumption (g/day)	7.1 ± 20.1	8.6 ± 23.2	9.9 ± 25.7	12.6 ± 30.6	<0.001
Healthy Eating Index-2015	71.4 ± 8.9	67.8 ± 9.0	65.2 ± 9.1	61.8 ± 9.1	<0.001
Energy intake from diet (kcal/day)	1407.8 ± 567.3	1,516.4 ± 580.2	1,754.5 ± 629.3	2,275.9 ± 816.8	<0.001
History of diabetes	1,401 (5.5%)	1,573 (6.2%)	1,725 (6.8%)	2,104 (8.3%)	<0.001
Family history of pancreatic Cancer	696 (2.7%)	699 (2.7%)	687 (2.7%)	518 (2.0%)	<0.001
Aspirin user	11,655 (45.8)	11,702 (46.0)	12,118 (47.6)	12,319 (48.4)	<0.001
**Food consumption**					
Fruits (g/day)	320.9 ± 245.2	266.5 ± 202.9	257.7 ± 203.4	250.5 ± 213.7	<0.001
Vegetables (g/day)	282.9 ± 207.7	255.6 ± 167.7	271.8 ± 169.9	325.8 ± 193.5	<0.001
Whole grain (servings/day)	1.2 ± 0.8	1.1 ± 0.8	1.1 ± 0.8	1.3 ± 0.9	<0.001
Red processed meat (g/day)	4.7 ± 8.2	8.7 ± 10.6	13.3 ± 13.9	22.9 ± 22.3	<0.001
Coffee (g/day)	694.2 ± 698.4	795.7 ± 746.4	880.3 ± 795.4	1015.3 ± 893.3	<0.001
Dairy (servings/day)	1.3 ± 1.1	1.3 ± 1.1	1.4 ± 1.1	1.5 ± 1.2	<0.001
Nuts (g/day)	5.9 ± 14.8	5.9 ± 13.0	6.7 ± 13.9	8.4 ± 16.1	<0.001

^a^Values are mean ± standard deviation or counts (percentage) as indicated.

^b^“Others” refers to Asian, Pacific Islander, or American Indian.

^c^Weight (kg)/height (m)^2^.

^d^Total time of moderate-to-vigorous physical activity per week.

### Fried food consumption and the risk of pancreatic cancer

During an average (standard deviation) follow-up of 8.86 (1.91) years (900871.2 person-years), a total of 402 pancreatic cancer cases were observed, with the overall incidence rate of 4.46 cases per 10,000 person-years. The results of Cox regression in the whole study population are summarized in Table 2. In the fully adjusted model (model 2), participants in the highest quartile of total fried food consumption were found to have a 29% lower risk of pancreatic cancer than those in the lowest quartile (HR_quartile 4 vs. 1_ 0.71, 95% CI 0.51–0.99, *P*_trend_ = 0.047, *E*-value = 2.17). Similar results were obtained for deep-fried foods (HR_quartile 4 vs. 1_ 0.64, 95% CI 0.47–0.88, *P*_trend_ = 0.011, *E*-value = 2.50). However, no significant association was found for pan-fried food consumption and the risk of pancreatic cancer (HR_quartile 4 vs. 1_ 0.98, 95% CI 0.73–1.32, *P*_trend_ = 0.815, *E*-value = 1.16), which did not alter materially when the association was investigated by doneness degree (Supplementary Table 4). When Cox regression analyses were repeated in 97,822 participants with complete covariate data, similar results were obtained (Supplementary Table 5).

**TABLE 2:** Hazard ratios of the association between energy-adjusted fried food consumption and the risk of pancreatic cancer.

Quartile of energy-adjusted fried food consumption (g/day)	Number of cases	Person-years	Crude incidence rate per 10,000 person-years	Hazard ratio (95% confidence interval)		
	
				Unadjusted	Model 1^a^	Model 2^b^
**Total fried foods**						
<6.10	106	227785.6	4.65	1.00 (reference)	1.00 (reference)	1.00 (reference)
6.10–15.06	98	225485.2	4.35	0.94 (0.71–1.23)	0.92 (0.70–1.22)	0.89 (0.68–1.18)
15.07–31.83	114	224597.6	5.08	1.09 (0.84–1.42)	1.06 (0.81–1.39)	1.00 (0.76–1.31)
>31.83	84	223002.8	3.77	0.81 (0.61–1.08)	0.78 (0.58–1.06)	0.71 (0.51–0.99)
*P*_trend_				0.171	0.116	0.047
**Deep-fried foods**						
<3.39	117	227376.1	5.15	1.00 (reference)	1.00 (reference)	1.00 (reference)
3.39–9.47	97	225228.3	4.31	0.84 (0.64–1.10)	0.83 (0.64–1.09)	0.81 (0.62–1.07)
9.48–21.60	107	224773.1	4.76	0.93 (0.71–1.20)	0.91 (0.69–1.19)	0.87 (0.67–1.15)
>21.60	81	223493.7	3.62	0.71 (0.53–0.94)	0.68 (0.51–0.92)	0.64 (0.47–0.88)
*P*_trend_				0.030	0.023	0.011
**Pan-fried foods**						
<0.56	98	227399.0	4.31	1.00 (reference)	1.00 (reference)	1.00 (reference)
0.56–2.95	88	225599.6	3.90	0.91 (0.68–1.21)	0.90 (0.68–1.20)	0.87 (0.65–1.16)
2.96–8.66	111	225124.1	4.93	1.15 (0.87–1.50)	1.11 (0.85–1.46)	1.06 (0.81–1.40)
>8.66	105	222748.5	4.71	1.10 (0.83–1.44)	1.05 (0.80–1.40)	0.98 (0.73–1.32)
*P*_trend_				0.316	0.494	0.815

^a^Adjusted for age (years) and sex (male, female).

^b^Adjusted for age (years), sex (male, female), smoking status [current (> 60 pack-years, 30–60 pack-years, < 30 pack-years), former (> 60 pack-years, 30–60 pack-years, < 30 pack-years), never], alcohol consumption (g/day), body mass index (kg/m^2^), aspirin use (yes, no), history of diabetes (yes, no), family history of pancreatic cancer (yes, no), and energy intake from diet (kcal/day).

**FIGURE 3:**
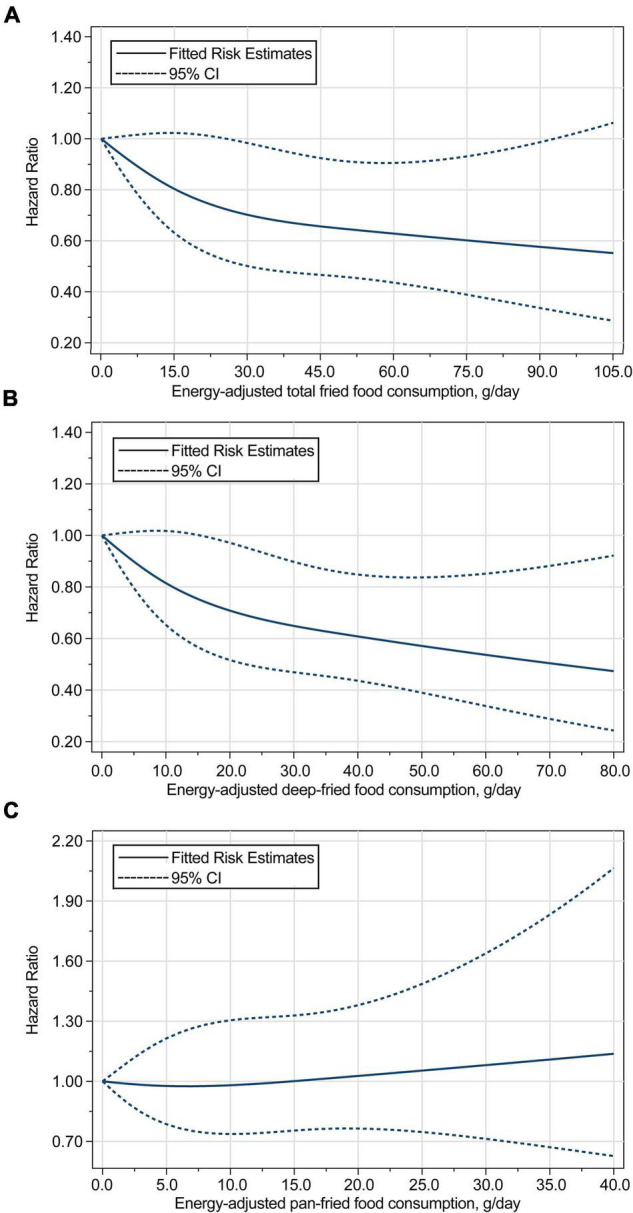
Dose–response analyses on the associations of energy-adjusted consumption of **(A)** total fried foods, **(B)** deep-fried foods, and **(C)** pan-fried foods with the risk of pancreatic cancer, with the reference level set at 0 g/day. Hazard ratios were adjusted for age, sex, smoking status, alcohol consumption, BMI, aspirin use, history of diabetes, family history of pancreatic cancer, and energy intake from diet. The P for non-linearity were 0.043 for total fried food consumption, 0.013 for deep-fried food consumption, and 0.900 for pan-fried food consumption.

Based on restricted cubic spline regression models, consumption of total fried foods (*P*_non–linearity_ = 0.043) and deep-fried foods (*P*_non–linearity_ = 0.013) was found to be inversely related to the risk of pancreatic cancer in a non-linear dose–response manner, whereas such a relationship was not found for pan-fried food consumption (*P*_non–linearity_ = 0.900) (Figure 3). The initial associations of total fried food, deep-fried food, and pan-fried food consumption with the risk of pancreatic cancer were found to be not modified by the predefined stratification factors (all *P*_interaction_ > 0.05) (Supplementary Table 6) and did not alter substantially in a series of sensitivity analyses (Supplementary Table 7).

### Explanatory analyses to identify the main contributor(s) to the observed association

**FIGURE 4:**
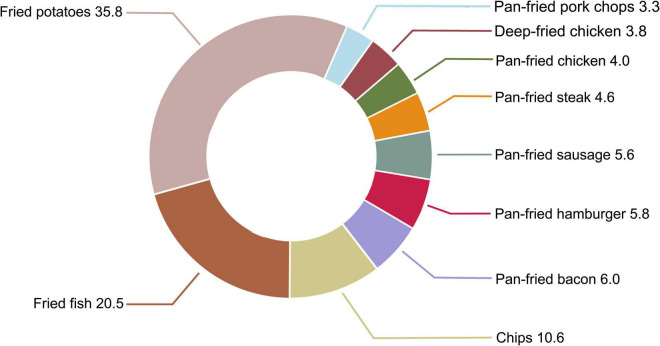
Proportion (%) of each fried food in total fried food consumption in our study population.

In this study population, main food items contributing to fried food consumption were fried potatoes (35.8%), followed by fried fish (20.5%) and chips (10.6%) (Figure 4). To identify the main contributor(s) to the observed association, we first examined the association between individual fried food consumption and the risk of pancreatic cancer. Among 10 fried foods, only chip consumption was found to be inversely associated with the risk of pancreatic cancer (HR_quartile 4 vs. 1_ 0.68, 95% CI 0.50–0.90, *P*_trend_ = 0.015) (Supplementary Table 8). We then examined the association of interest with further adjustment for individual fried food consumption. Consistently, only after further adjustment for chip consumption, the initial significant associations of consumption of total fried foods and deep-fried foods with the risk of pancreatic cancer changed to be non-significant (*P*_trend_ = 0.287 for total fried foods and *P*_trend_ = 0.133 for deep-fried foods, Supplementary Table 9). Finally, we examined the association of interest after ignoring an individual fried food in each turn. As expected, ignoring “chips” resulted in non-significant associations between consumption of total fried foods (*P*_trend_ = 0.122) and deep-fried foods (*P*_trend_ = 0.180) and the risk of pancreatic cancer, although ignoring “pan-fried chicken” (*P*_trend_ = 0.135) and “pan-fried steak” (*P*_trend_ = 0.161) also led to a similar phenomenon (Supplementary Table 10).

## Discussion

Interestingly, contrary to our initial hypothesis, consumption of total fried foods and deep-fried foods was found to be inversely associated with the risk of pancreatic cancer in a non-linear dose–response manner, while no significant association was found for pan-fried food consumption and the risk of pancreatic cancer. Moreover, these observations were not modified by the predefined stratification factors and remained in a series of sensitivity analyses.

A growing number of evidence has suggested an important role of dietary habits in the etiology of pancreatic cancer (11). For example, a 2017 systematic review on dietary pattern and pancreatic cancer found that unfavorable patterns (e.g., western dietary pattern) were positively associated with while favorable patterns (e.g., prudent dietary pattern) were inversely associated with the risk of pancreatic cancer (33). However, whether fried food consumption is associated with the risk of pancreatic cancer remains to be elucidated. A1994 follow-up study of 9,990 Finnish men and women with only 29 pancreatic cancer cases revealed a null association between fried meat consumption and the risk of pancreatic cancer (relative risk for the highest vs. the lowest tertiles of fried meat consumption: 1.54; 95% CI: 0.53, 4.50) (34), whereas a later hospital-based case-control study of 629 patients in Iran found that deep-fried vegetable consumption conferred an increased risk of pancreatic cancer (odds ratio for consumers vs. non-consumers: 1.70; 95% CI: 1.16, 2.48) (35). Importantly, these studies had an inability to consider the potential antagonistic or synergistic effects among individual fried foods. A 1995 population-based case-control study of 418 individuals in Canada observed that more frequent consumption of fried foods conferred a higher risk of pancreatic cancer (relative risk for often vs. never: 3.84; 95% CI: 1.74, 8.48) (15). By contrast, based on prospective data of 101,729 American adults, our study revealed inverse associations of total fried food and deep-fried food consumption with the risk of pancreatic cancer. Compared with some of previous studies in this field (15, 34, 35), our study has several advantages. First, our study design is a prospective study, which allows to establish a temporal association and make our results be free of recall bias. Second, we used dietary pattern approach to evaluate the association of fried food consumption with the risk of pancreatic cancer, enabling our results to account for the potential interactions among individual fried foods (36). Third, our study had a larger sample size and a longer follow-up length, thereby producing more pancreatic cancer cases, which make our study have higher power to detect the potential association of fried food consumption with the risk of pancreatic cancer. Lastly, we explored the potential dose–response associations between fried food consumption and the risk of pancreatic cancer using the well-developed method; the corresponding results provided a detailed description for the risk of pancreatic cancer across the indicated range of fried food consumption.

Consumers have own preferences for the degree of meat doneness in their daily life. A recent study found that well-done pork steak had higher energy content but lower retention ratios for iron and potassium than medium- or rare-done pork steak (37). In addition, another study found that percent fat and protein in beef increased with increase in the doneness degree (38). Moreover, it was found that the contents of heterocyclic aromatic amines, a class of mutagenic compounds formed during cooking of meats, also increased with increase in the doneness degree (39). Thus, doneness degree may impact the association of meat consumption with cancer risk. Indeed, observational studies have found that well-done meat consumption has a stronger positive association with the risk of colorectal cancer than rare- or medium-done meat consumption (40, 41). Given the above facts, we investigated the association of pan-fried food consumption with the risk of pancreatic cancer by doneness degree; nevertheless, the corresponding results indicated that the doneness degree of pan-fried foods had little influence on this association. The exact reasons behind this observation are unclear. One possible explanation is that doneness degree of fried foods does play minimal roles in the etiology of pancreatic cancer; also, another explanation may be that pan-fried food consumption by doneness is so small that the current study is incapable of detecting the potential influence of doneness on the association of interest. Of note, the association between fried food consumption and the risk of pancreatic cancer may be also affected by other factors, such as the type of oil used, whether the oil is reused, and the location where fried foods are eaten (away from home vs. at home). Unfortunately, in the PLCO Cancer Screening Trial, the information on these important factors was not collected, which precluded us to perform the relevant analyses to explore their potential impacts on the association of fried food consumption with the risk of pancreatic cancer. Nevertheless, in the US, fried foods are most often consumed at fast food restaurants, where corn oil is the most common frying medium and is frequently reused (26, 42). Overall, to obtain a better understanding for the association of fried food consumption with the risk of pancreatic cancer, more studies are needed to clarify the potential influence of these factors.

Interestingly, we observed that consumption of deep-fried foods, but not pan-fried foods, was inversely related to the risk of pancreatic cancer in our study population. The specific reasons for the differential association of deep-fried foods and pan-fried foods with the risk of pancreatic cancer are unknown. Considering the fundamental difference between deep-frying and pan-frying (i.e., foods are completely immersed in frying medium in deep-frying while only partially immersed in frying medium in pan-frying), the above-mentioned phenomenon may be due to that foods absorb different amounts of fat, such as saturated fatty acids and monounsaturated fatty acids. Indeed, a recent large prospective cohort study found that high dietary intakes of saturated fatty acids conferred an increased risk of pancreatic cancer (HR_quartile 4 vs. 1_ 1.05, 95% CI 1.01–1.09, *P*_trend_ = 0.01) while high dietary intakes of monounsaturated fatty acids exerted opposite effect (HR_quartile 4vs. 1_ 0.92, 95% CI 0.86–0.99, *P*_trend_ = 0.04) (43). In addition, we noticed that deep-fried food consumption was much higher than pan-fried food consumption in our study population (the proportion in total fried food consumption: 70.7% for deep-fried foods vs. 29.3% for pan-fried foods, Figure 4). Thus, another explanation is that for a given participant, the potential influence of pan-fried food consumption on the risk of pancreatic cancer has been masked by the influence of deep-fried food consumption.

The inverse associations of total fried food and deep-fried food consumption with the risk of pancreatic cancer may be accounted by several mechanisms. Intuitively, the beneficial role of total fried foods or deep-fried foods in reducing the risk of pancreatic cancer may be attributable to individual fried foods. Consistent with this speculation, our explanatory analyses indicated that chips could be the main contributors to the observed associations. Potato chips are good sources of antioxidants; an animal study found that feeding potato chips elevated ascorbic acid levels and thus decreased reactive oxygen species levels in mouse tissues (44). Functionally, antioxidants protect cells from oxidative DNA damage, which plays a critical role in the initiation of pancreatic cancer (45); our recent work also showed that dietary antioxidant capacity was inversely related to the risk of pancreatic cancer (46). Meanwhile, potato chips contain abundant amounts of folate and magnesium, whose consumption has been inversely associated with the risk of pancreatic cancer (47, 48). In addition, a randomized crossover trial found that tortilla and corn chips could reduce serum levels of LDL cholesterol (49), which has been found to promote the proliferation of pancreatic cancer cells through activating STAT3 pathway (50). Nevertheless, it should be noted that the observed associations may be also partly attributable to the potential interactions between individual fried foods, given dietary pattern approach we used. In addition, in our study population, participants in the highest vs. the lowest quartiles of total fried food and deep-fried food consumption were found to consume more vegetables and coffee; therefore, it is possible that the inverse associations of total fried food and deep-fried food consumption with the risk of pancreatic cancer are actually mediated by these two factors, considering that both vegetable and coffee consumption have been inversely associated with the risk of pancreatic cancer (14, 51). However, the observation that the initial results did not change materially after adjusting for these two factors (Supplementary Table 6), makes this unlikely.

Our study has several limitations. First, dietary assessment was performed once at baseline in this study, and thus may be subject to non-differential bias, given that dietary habits could change over time. Nonetheless, it has been demonstrated that the approach only using baseline diet generally results in a weaker association than does that using the cumulative averages (52). In addition, like other food frequency questionnaires, data collection by the DHQ might be subject to recall bias. Nevertheless, the DHQ had been validated against four 24-h dietary recalls (21), which may attenuate this concern. Second, because the public perceives fried foods as being unhealthy, the possibility of under-reporting cannot be excluded. Nevertheless, under-reporting bias is non-differential, as this bias is not anticipated to be related to the future pancreatic cancer risk, and thus would bias risk estimates toward null, indicating that the true association between fried food consumption and the risk of pancreatic cancer would be stronger than that we observed. Third, as different countries or regions have different cooking traditions, which result in different frying practices and media, therefore, our findings in this US population may not be applicable to other populations. Fourth, as with any observational study, our results might be susceptible to residual confounding because of unrecognized or unmeasured confounders, though we had controlled for the potential confounders. Thus, we cannot rule out the possibility that our results were due to the lack of adjustment for variables associated with the risk of pancreatic cancer, such as serum glucose and lipids. Moreover, our results cannot establish a causal association of fried food consumption with the risk of pancreatic cancer, considering the observational nature of our study.

## Conclusion

In conclusion, in this prospective multicenter cohort study of 101,729 US adults, high consumption of deep-fried foods, but not pan-fried foods, is found to be associated with a reduced risk of pancreatic cancer in a non-linear dose–response manner. The role of deep-fried foods in decreasing the risk of pancreatic cancer appears to be mainly attributable to chips. Our findings provide a novel and unique insight into the role of fried foods in the etiology of pancreatic cancer and may alleviate the people’s concern that fried foods are potentially carcinogenic. Given the high popularity of fried foods, our findings have important public health implications. More studies are needed to confirm our findings in other populations and settings and to clarify the underlying biological mechanisms.

## Data availability statement

The datasets presented in this article are not readily available because US National Cancer Institute’s data policy. Requests to access the datasets should be directed to US National Cancer Institute.

## Ethics statement

The studies involving human participants were reviewed and approved by Institutional Review Boards of the US National Cancer Institute and each screening center. The patients/participants provided their written informed consent to participate in this study.

## Author contributions

G-CZ and J-HG developed the hypothesis, study design, and concept and other authors made useful suggestions and acted as guarantors for the integrity of the data and the accuracy of statistical analysis. G-CZ applied and acquired the original data from the US National Cancer Institute, drafted study protocol and the initial manuscript, and other authors made critical comments and revisions, and responsible for manuscript submission and had full access to the original data. QZ was responsible for statistical analyses. G-CZ attested that all listed authors meet authorship criteria and that no others meeting the criteria have been omitted. All authors interpreted the corresponding results together and approved the final version of the article, including the authorship list.

## Conflict of interest

The authors declare that the research was conducted in the absence of any commercial or financial relationships that could be construed as a potential conflict of interest.

## Publisher’s note

All claims expressed in this article are solely those of the authors and do not necessarily represent those of their affiliated organizations, or those of the publisher, the editors and the reviewers. Any product that may be evaluated in this article, or claim that may be made by its manufacturer, is not guaranteed or endorsed by the publisher.

## Footnotes

1
www.evalue-calculator.com/
