# Supplementary material for: Fried food consumption and the risk of pancreatic cancer: A large prospective multicenter study
Source: Front Nutr. 2022 Jul 22;9:889303. doi: 10.3389/fnut.2022.889303 (PMC9362838; doi:10.3389/fnut.2022.889303)
Supplement: Supplementary file 1 [file Data_Sheet_1.DOCX]

| **Supplemental Table 1.** Sociodemographic characteristics of study population by study center ^a^ | | | | | | | | | | |
| --- | --- | --- | --- | --- | --- | --- | --- | --- | --- | --- |
| Characteristics | Study centers | | | | | | | | | |
|  | University of Colorado | Georgetown University | Pacific Health Research and Education Institute | Henry Ford Health System | University of Minnesota | Washington University | University of Pittsburgh | University of Utah | Marshfield Clinic Research  Foundation | University of Alabama |
| No. of subjects | 8701 | 4818 | 3711 | 14177 | 20950 | 10342 | 12093 | 10241 | 12796 | 3900 |
| Age (years) | 65.2 ± 5.5 | 67.0 ± 5.6 | 66.4 ± 6.1 | 64.2 ± 5.9 | 66.3 ± 5.7 | 65.3 ± 5.6 | 65.0 ± 5.8 | 66.4 ± 5.6 | 65.7 ± 5.5 | 63.7 ± 5.3 |
| Male | 5083 (58.4) | 2728 (56.6) | 2022 (54.5) | 5717 (40.3) | 12314 (58.8) | 4284 (41.4) | 5735 (47.4) | 3695 (36.1) | 6564 (51.3) | 1339 (34.3) |
| Ethnic group | | | | | | | | | | |
| Non-Hispanic white | 7714 (88.7) | 4322 (89.7) | 147 (4.0) | 12480 (88.0) | 20639 (98.5) | 9901 (95.7) | 11615 (96.0) | 10012 (97.8) | 12702 (99.3) | 2980 (76.4) |
| Non-Hispanic black | 134 (1.5) | 304 (6.3) | 2 (0.1) | 1288 (9.1) | 72 (0.3) | 308 (3.0) | 350 (2.9) | 18 (0.2%) | 0 (0.0%) | 874 (22.4) |
| Hispanic | 705 (8.1) | 84 (1.7) | 12 (0.3) | 202 (1.4) | 141 (0.7) | 74 (0.7) | 72 (0.6) | 118 (1.2) | 60 (0.5) | 27 (0.7) |
| Others ^b^ | 148 (1.7) | 108 (2.2) | 3550 (95.7) | 207 (1.5) | 98 (0.5) | 59 (0.6) | 56 (0.5) | 93 (0.9) | 34 (0.3) | 19 (0.5) |
| Educational degree | | | | | | | | | | |
| College below | 4313 (49.6) | 1552 (32.2) | 2217 (59.7) | 9349 (65.9) | 13413 (64.0) | 6724 (65.0) | 7858 (65.0) | 6906 (67.4) | 9923 (77.5) | 2680 (68.7) |
| College graduate | 2027 (23.3) | 1227 (25.5) | 772 (20.8) | 2262 (16.0) | 3936 (18.8) | 1759 (17.0) | 2031 (16.8) | 1746 (17.0) | 1449 (11.3) | 633 (16.2) |
| Postgraduate | 2361 (27.1) | 2039 (42.3) | 722 (19.5) | 2566 (18.1) | 3601 (17.2) | 1859 (18.0) | 2204 (18.2) | 1589 (15.5) | 1424 (11.1) | 587 (15.1) |
| Body mass index | 26.3 ± 4.3 | 26.1 ± 4.1 | 25.2 ± 4.0 | 27.7 ± 5.3 | 27.3 ± 4.5 | 27.1 ± 4.8 | 27.5 ± 4.9 | 27.0 ± 4.7 | 28.0 ± 4.8 | 27.9 ± 5.4 |
| Smoking status | | | | | | | | | | |
| Current | 815 (9.4) | 317 (6.6) | 278 (7.5) | 1765 (12.4) | 1833 (8.7) | 1084 (10.5) | 1194 (9.9) | 612 (6.0) | 1111 (8.7) | 390 (10.0) |
| Former | 4229 (48.6) | 2287 (47.5) | 1505 (40.6) | 6221 (43.9) | 9923 (47.4) | 4648 (44.9) | 5113 (42.3) | 2859 (27.9) | 5565 (43.5) | 1410 (36.2) |
| Never | 3657 (42.0) | 2214 (46.0) | 1928 (52.0) | 6191 (43.7) | 9194 (43.9) | 4610 (44.6) | 5786 (47.8) | 6770 (66.1) | 6120 (47.8) | 2100 (53.8) |
| Alcohol consumption (g/day) | 11.5 ± 25.7 | 12.9 ± 25.0 | 7.4 ± 23.7 | 8.5 ± 24.0 | 11.1 ± 26.9 | 9.7 ± 26.6 | 9.1 ± 25.6 | 4.7 ± 17.9 | 11.7 ± 27.8 | 5.5 ± 21.1 |
| History of diabetes | 497 (5.7) | 257 (5.3) | 369 (9.9) | 1177 (8.3) | 1162 (5.5) | 637 (6.2) | 839 (6.9) | 631 (6.2) | 846 (6.6) | 388 (9.9) |
| Family history of pancreatic cancer | 209 (2.4) | 132 (2.7) | 122 (3.3) | 337 (2.4) | 498 (2.4) | 253 (2.4) | 319 (2.6) | 248 (2.4) | 373 (2.9) | 109 (2.8) |

^a^ Values are mean ± standard deviation or counts (percentage) as indicated.

^b^ “Others” refers to Asian, Pacific Islander, or American Indian.

| **Supplemental Table 2.** Comparison of sociodemographic characteristics between included and excluded populations ^a^ | | | |
| --- | --- | --- | --- |
| Characteristics | Included population | Excluded population | Standardized  difference |
| Number of participants | 101729 | 53159 |  |
| Age (years) | 65.5 ± 5.7 | 66.8 ± 5.9 | 0.22 |
| Sex | | | |
| Male | 49481 (48.6) | 27197 (51.2) | 0.05 |
| Female | 52248 (51.4) | 25961 (48.8) |  |
| Body mass index (kg/m^2^) | 27.2 ± 4.8 | 27.4 ± 5.1 | 0.03 |
| Educational level | | | |
| College below | 64935 (63.8) | 32259 (67.2) | 0.07 |
| College graduate | 17842 (17.6) | 7500 (15.6) |  |
| Postgraduate | 18952 (18.6) | 8275 (17.2) |  |
| Occupation | | | |
| Retired | 43704 (43.2) | 20804 (43.4) | 0.10 |
| Working | 40716 (40.2) | 18509 (38.6) |  |
| Homemaker | 11855 (11.7) | 5131 (10.7) |  |
| Other | 4988 (4.9) | 3501 (7.3) |  |
| Smoking status | | | |
| Current | 9399 (9.2) | 6654 (13.8) | 0.15 |
| Past | 43760 (43.1) | 20855 (43.2) |  |
| Never | 48570 (47.7) | 20718 (43.0) |  |
| Pack-years | 17.8 ± 26.7 | 21.9 ± 30.3 | 0.14 |
| Alcohol consumption (g/day) | 9.6 ± 25.3 | 19.34 ± 80.0 | 0.17 |
| History of diabetes | 6803 (6.7) | 4726 (9.9) | 0.11 |
| Family history of pancreatic cancer | 2600 (2.6) | 1199 (2.5) | 0.04 |
| Trial group | | | |
| Intervention group | 51804 (50.9) | 25639 (48.2) | 0.05 |
| Control group | 49925 (49.1) | 27519 (51.8) |  |

^a^ Values are mean ± standard deviation or counts (percentage) as indicated.

| **Supplemental Table 3.** Distribution of variables with missing values before and after imputation ^a^ | | | |
| --- | --- | --- | --- |
| Variables | Before imputation | After imputation | Number (%) with missing data |
| Educational level | | | |
| College below | 64731 (63.8) | 64935 (63.8) | 204 (0.20) |
| College graduate | 17842 (17.6) | 17842 (17.5) |  |
| Postgraduate | 18952 (18.7) | 18952 (18.6) |  |
| History of diabetes | | | |
| Yes | 6803 (6.7) | 6803 (6.7) | 538 (0.53) |
| No | 94388 (93.3) | 94926 (93.3) |  |
| Family history of pancreatic cancer | | | |
| Yes | 2600 (2.6) | 2600 (2.6) | 781 (0.77) |
| No | 95719 (94.8) | 96500 (94.9) |  |
| Possibly | 2629 (2.6) | 2629 (2.6) |  |
| Smoking status | | | |
| Current | 9399 (9.2) | 9399 (9.2) | 20 (0.02) |
| Past | 43760 (43.0) | 43760 (43.0) |  |
| Never | 48550 (47.7) | 48570 (47.7) |  |
| Pack-years (packs smoked/year) | 17.8 ± 26.7 | 17.7 ± 26.6 | 1164 (1.14) |
| Body mass index (kg/m^2^) | 27.2 ± 4.8 | 27.2 ± 4.8 | 1348 (1.33) |
| Physical activity level (min/week) ^b^ | 124.4 ± 122.6 | 122.0 ± 122.8 | 26138 (25.69) |
| Aspirin use | | | |
| Yes | 47794 (47.2) | 47794 (47.0) | 443 (0.44) |
| No | 53492 (52.8) | 53935 (53.0) |  |

^a^ Values are mean (standard deviation) or counts (percentage) as indicated.

^b^ Total time of moderate-to-vigorous physical activity per week.

| **Supplemental Table 4.** Hazard ratios of the association of energy-adjusted pan-fried food consumption with the risk of pancreatic cancer by doneness degree | | | | | | |
| --- | --- | --- | --- | --- | --- | --- |
| Quartile of energy-adjusted pan-fried food consumption (g/day) | Number of cases | Person-years | Crude incidence rate per 10000 person-years | Hazard ratio (95% confidence interval) | | |
|  |  |  |  | Unadjusted | Model 1^a^ | Model 2 ^b^ |
| Just done | | | | | | |
| <-0.34 | 103 | 226150.2 | 4.55 | 1.00 (reference) | 1.00 (reference) | 1.00 (reference) |
| -0.33–-0.06 | 90 | 226472.0 | 3.97 | 0.87 (0.66 to 1.16) | 0.93 (0.69 to 1.23) | 0.83 (0.60 to 1.14) |
| -0.07–1.97 | 94 | 225116.4 | 4.18 | 0.92 (0.69 to 1.21) | 0.95 (0.72 to 1.26) | 0.84 (0.62 to 1.15) |
| >1.98 | 115 | 223133.0 | 5.15 | 1.13 (0.87 to 1.48) | 1.11 (0.85 to 1.45) | 1.02 (0.78 to 1.34) |
| *P*_trend_ | | | | 0.087 | 0.209 | 0.362 |
| Well done | | | | | | |
| <-0.01 | 101 | 226529.2 | 4.46 | 1.00 (reference) | 1.00 (reference) | 1.00 (reference) |
| -0.01–1.32 | 102 | 225714.1 | 4.52 | 1.01 (0.77 to 1.33) | 1.04 (0.79 to 1.37) | 1.01 (0.76 to 1.33) |
| 1.33–4.91 | 91 | 225083.4 | 4.04 | 0.91 (0.68 to 1.20) | 0.91 (0.68 to 1.21) | 0.87 (0.66 to 1.16) |
| >4.91 | 108 | 223545.0 | 4.83 | 1.09 (0.83 to 1.42) | 1.07 (0.82 to 1.41) | 1.01 (0.77 to 1.34) |
| *P*_trend_ | | | | 0.467 | 0.582 | 0.847 |
| Very well done | | | | | | |
| <-0.23 | 99 | 224555.4 | 4.41 | 1.00 (reference) | 1.00 (reference) | 1.00 (reference) |
| -0.23–-0.15 | 99 | 226240.5 | 4.38 | 0.99 (0.75 to 1.31) | 1.04 (0.78 to 1.37) | 1.02 (0.73 to 1.41) |
| -0.16–-0.08 | 99 | 225707.8 | 4.39 | 0.99 (0.75 to 1.31) | 1.09 (0.82 to 1.45) | 1.04 (0.71 to 1.53) |
| >-0.08 | 105 | 224367.9 | 4.68 | 1.06 (0.81 to 1.40) | 1.15 (0.87 to 1.53) | 1.07 (0.75 to 1.54) |
| *P*_trend_ | | | | 0.592 | 0.321 | 0.662 |

^a^ Adjusted for age (years) and sex (male, female).

^b^ Adjusted for age (years), sex (male, female), smoking status [current (>60 pack-years, 30–60 pack-years, <30 pack-years), former (>60 pack-years, 30–60 pack-years, <30 pack-years), never], alcohol consumption (g/day), body mass index (kg/m^2^), aspirin use (yes, no), history of diabetes (yes, no), family history of pancreatic cancer (yes, no), and energy intake from diet (kcal/day).

| **Supplemental Table 5.** Hazard ratios of the association between energy-adjusted fried food consumption and the risk of pancreatic cancer in 97822 participants with complete covariate data | | | | | | |
| --- | --- | --- | --- | --- | --- | --- |
| Quartile of energy-adjusted fried food consumption (g/day) | Number of cases | Person-years | Crude incidence rate per 10000 person-years | Hazard ratio (95% confidence interval) | | |
|  |  |  |  | Unadjusted | Model 1^a^ | Model 2 ^b^ |
| Total fried foods | | | | | | |
| <6.10 | 104 | 219168.2 | 4.75 | 1.00 (reference) | 1.00 (reference) | 1.00 (reference) |
| 6.10–15.06 | 94 | 217647.1 | 4.32 | 0.91 (0.69 to 1.20) | 0.90 (0.68 to 1.20) | 0.88 (0.66 to 1.16) |
| 15.07–31.83 | 107 | 215855.0 | 4.96 | 1.05 (0.80 to 1.37) | 1.02 (0.77 to 1.34) | 0.96 (0.73 to 1.28) |
| >31.83 | 82 | 214227.5 | 3.83 | 0.81 (0.61 to 1.08) | 0.78 (0.58 to 1.06) | 0.72 (0.52 to 1.01) |
| *P*_trend_ | | | | 0.187 | 0.133 | 0.071 |
| Deep-fried foods | | | | | | |
| <3.39 | 113 | 218631.4 | 5.17 | 1.00 (reference) | 1.00 (reference) | 1.00 (reference) |
| 3.39–9.47 | 92 | 217314.3 | 4.23 | 0.82 (0.62 to 1.08) | 0.82 (0.62 to 1.08) | 0.80 (0.60 to 1.05) |
| 9.48–21.60 | 103 | 216246.5 | 4.76 | 0.92 (0.71 to 1.20) | 0.91 (0.69 to 1.19) | 0.88 (0.67 to 1.16) |
| >21.60 | 79 | 214705.5 | 3.68 | 0.71 (0.53 to 0.95) | 0.69 (0.51 to 0.94) | 0.66 (0.48 to 0.91) |
| *P*_trend_ | | | | 0.046 | 0.037 | 0.025 |
| Pan-fried foods | | | | | | |
| <0.56 | 96 | 219200.9 | 4.38 | 1.00 (reference) | 1.00 (reference) | 1.00 (reference) |
| 0.56–2.95 | 86 | 217177.9 | 3.96 | 0.90 (0.68 to 1.21) | 0.90 (0.67 to 1.20) | 0.87 (0.65 to 1.16) |
| 2.96–8.66 | 105 | 216626.0 | 4.85 | 1.11 (0.84 to 1.46) | 1.08 (0.82 to 1.42) | 1.03 (0.78 to 1.36) |
| >8.66 | 100 | 213893.0 | 4.68 | 1.07 (0.81 to 1.42) | 1.03 (0.77 to 1.37) | 0.97 (0.72 to 1.30) |
| *P*_trend_ | | | | 0.419 | 0.616 | 0.900 |

^a^ Adjusted for age (years) and sex (male, female).

^b^ Adjusted for age (years), sex (male, female), smoking status [current (>60 pack-years, 30–60 pack-years, <30 pack-years), former (>60 pack-years, 30–60 pack-years, <30 pack-years), never], alcohol consumption (g/day), body mass index (kg/m^2^), aspirin use (yes, no), history of diabetes (yes, no), family history of pancreatic cancer (yes, no), and energy intake from diet (kcal/day).

| **Supplemental Table 6.** Subgroup analyses on the association between energy-adjusted fried food consumption and the risk of pancreatic cancer ^a^ | | | | | | |
| --- | --- | --- | --- | --- | --- | --- |
| Subgroup variable | Total fried food consumption and pancreatic cancer | | Deep-fried food consumption and pancreatic cancer | | Pan-fried food consumption and pancreatic cancer | |
|  | Quartile 1 | Quartile 4 | Quartile 1 | Quartile 4 | Quartile 1 | Quartile 4 |
| **Age (years)** | | | | | | |
| ≥65 |  |  |  |  |  |  |
| Cases (person-years) | 83 (138503.68) | 56 (101282.80) | 93 (140715.6) | 51 (100985.28) | 69 (127340.60) | 68 (112104.32) |
| HR (95% CI) | 1.00 (reference) | 0.77 (0.52 to 1.14) | 1.00 (reference) | 0.63 (0.43 to 0.93) | 1.00 (reference) | 1.01 (0.70 to 1.44) |
| <65 |  |  |  |  |  |  |
| Cases (person-years) | 23 (89276.25) | 28 (121727.77) | 24 (86655.42) | 30 (122494.24) | 29 (100060.65) | 37 (110668.80) |
| HR (95% CI) | 1.00 (reference) | 0.65 (0.35 to 1.21) | 1.00 (reference) | 0.71 (0.39 to 1.27) | 1.00 (reference) | 0.92 (0.55 to 1.55) |
| *P*_interaction_ |  | 0.343 |  | 0.658 |  | 0.879 |
| **Sex** | | | | | | |
| Male |  |  |  |  |  |  |
| Cases (person-years) | 44 (66304.06) | 69 (156048.75) | 51 (68790.54) | 60 (152765.64) | 46 (83540.63) | 84 (142117.64) |
| HR (95% CI) | 1.00 (reference) | 0.74 (0.48 to 1.12) | 1.00 (reference) | 0.59 (0.39 to 0.88) | 1.00 (reference) | 1.12 (0.76 to 1.63) |
| Female |  |  |  |  |  |  |
| Cases (person-years) | 62 (161521.14) | 15 (66879.45) | 66 (158639.07) | 21 (70658.12) | 52 (143907.72) | 21 (80625.10) |
| HR (95% CI) | 1.00 (reference) | 0.61 (0.33 to 1.11) | 1.00 (reference) | 0.78 (0.46 to 1.32) | 1.00 (reference) | 0.71 (0.42 to 1.20) |
| *P*_interaction_ |  | 0.819 |  | 0.541 |  | 0.266 |
| **Body mass index** | | | | | | |
| ≥25 |  |  |  |  |  |  |
| Cases (person-years) | 62 (120770.66) | 67 (170677.5) | 67 (122221.84) | 63 (169269.62) | 56 (126086.87) | 79 (166184.28) |
| HR (95% CI) | 1.00 (reference) | 0.72 (0.49 to 1.07) | 1.00 (reference) | 0.66 (0.45 to 0.96) | 1.00 (reference) | 1.01 (0.70 to 1.45) |
| <25 |  |  |  |  |  |  |
| Cases (person-years) | 44 (106926.12) | 17 (52208.06) | 50 (105043.35) | 18 (54146.40) | 42 (101250.00) | 26 (56540.64) |
| HR (95% CI) | 1.00 (reference) | 0.64 (0.34 to 1.19) | 1.00 (reference) | 0.58 (0.32 to 1.05) | 1.00 (reference) | 0.92 (0.55 to 1.56) |
| *P*_interaction_ |  | 0.542 |  | 0.765 |  | 0.702 |
| **Aspirin use** | | | | | | |
| Yes |  |  |  |  |  |  |
| Cases (person-years) | 51 (103612.95) | 39 (107052.11) | 56 (104133.80) | 37 (106844.70) | 47 (104399.90) | 50 (106691.20) |
| HR (95% CI) | 1.00 (reference) | 0.60 (0.38 to 0.97) | 1.00 (reference) | 0.56 (0.35 to 0.88) | 1.00 (reference) | 0.92 (0.60 to 1.40) |
| No |  |  |  |  |  |  |
| Cases (person-years) | 55 (124139.78) | 45 (116050.05) | 61 (123237.00) | 44 (116649.37) | 51 (122967.00) | 55 (116041.20) |
| HR (95% CI) | 1.00 (reference) | 0.83 (0.53 to 1.31) | 1.00 (reference) | 0.73 (0.47 to 1.13) | 1.00 (reference) | 1.05 (0.70 to 1.58) |
| *P*_interaction_ |  | 0.478 |  | 0.728 |  | 0.795 |
| **Smoking status** | | | | | | |
| Current or past |  |  |  |  |  |  |
| Cases (person-years) | 53 (103580.40) | 54 (131424.16) | 58 (105496.02) | 53 (129365.07) | 46 (105629.16) | 69 (130386.24) |
| HR (95% CI) | 1.00 (reference) | 0.81 (0.52 to 1.24) | 1.00 (reference) | 0.75 (0.50 to 1.14) | 1.00 (reference) | 1.26 (0.85 to 1.86) |
| Never |  |  |  |  |  |  |
| Cases (person-years) | 53 (124110.16) | 30 (91688.64) | 59 (121786.88) | 28 (94073.45) | 52 (121760.52) | 36 (92345.13) |
| HR (95% CI) | 1.00 (reference) | 0.73 (0.44 to 1.21) | 1.00 (reference) | 0.58 (0.35 to 0.95) | 1.00 (reference) | 0.88 (0.56 to 1.38) |
| *P*_interaction_ |  | 0.429 |  | 0.317 |  | 0.353 |
| **Alcohol consumption** | | | | | | |
| ≥median |  |  |  |  |  |  |
| Cases (person-years) | 46 (99362.28) | 48 (126203.25) | 50 (100647.00) | 49 (126789.97) | 47 (102647.82) | 53 (121319.58) |
| HR (95% CI) | 1.00 (reference) | 0.69 (0.43 to 1.10) | 1.00 (reference) | 0.70 (0.45 to 1.09) | 1.00 (reference) | 0.83 (0.55 to 1.27) |
| <median |  |  |  |  |  |  |
| Cases (person-years) | 60 (128348.55) | 36 (96741.97) | 67 (126825.00) | 32 (96667.29) | 51 (124733.50) | 52 (101413.60) |
| HR (95% CI) | 1.00 (reference) | 0.73 (0.45 to 1.16) | 1.00 (reference) | 0.58 (0.36 to 0.92) | 1.00 (reference) | 1.15 (0.77 to 1.74) |
| *P*_interaction_ |  | 0.472 |  | 0.381 |  | 0.516 |
| **Trial group** | | | | | | |
| Intervention group |  |  |  |  |  |  |
| Cases (person-years) | 56 (109399.68) | 37 (110771.05) | 62 (109074.57) | 38 (110800.62) | 53 (110061.63) | 49 (109990.08) |
| HR (95% CI) | 1.00 (reference) | 0.57 (0.35 to 0.91) | 1.00 (reference) | 0.56 (0.35 to 0.87) | 1.00 (reference) | 0.83 (0.55 to 1.25) |
| Control group |  |  |  |  |  |  |
| Cases (person-years) | 50 (118387.17) | 47 (112262.53) | 55 (118344.50) | 43 (112618.55) | 45 (117265.50) | 56 (112840.00) |
| HR (95% CI) | 1.00 (reference) | 0.88 (0.56 to 1.39) | 1.00 (reference) | 0.73 (0.47 to 1.14) | 1.00 (reference) | 1.17 (0.77 to 1.77) |
| *P*_interaction_ |  | 0.538 |  | 0.775 |  | 0.908 |

Abbreviations: HR, hazard ratio; CI, confidence interval.

^a^ HRs were adjusted for age (years), sex (male, female), smoking status [current (>60 pack-years, 30–60 pack-years, <30 pack-years), former (>60 pack-years, 30–60 pack-years, <30 pack-years), never], alcohol consumption (g/day), body mass index (kg/m^2^), aspirin use (yes, no), history of diabetes (yes, no), family history of pancreatic cancer (yes, no), and energy intake from diet (kcal/day). In subgroup analyses stratified by sex, smoking status, and aspirin use, hazard ratios were not adjusted for the stratification factor.

| **Supplemental Table 7.** Sensitivity analyses on the association between fried food consumption and the risk of pancreatic cancer ^a^ | | | | | |
| --- | --- | --- | --- | --- | --- |
| Categories | Quartiles of fried food consumption | | | | *P*_trend_ |
|  | Quartile 1 | Quartile 2 | Quartile 3 | Quartile 4 |  |
| **Total fried food consumption and pancreatic cancer** | | | | | |
| Excluded participants with extreme values of energy intake ^b^ | 1.00 (reference) | 0.91 (0.69 to 1.21) | 1.00 (0.76 to 1.33) | 0.71 (0.51 to 0.99) | 0.042 |
| Excluded participants with extreme total fried food consumption ^c^ | 1.00 (reference) | 0.82 (0.62 to 1.09) | 0.91 (0.69 to 1.20) | 0.63 (0.45 to 0.89) | 0.014 |
| Excluded cases occurred within the first two years of follow-up | 1.00 (reference) | 0.91 (0.67 to 1.24) | 1.04 (0.77 to 1.41) | 0.75 (0.53 to 1.07) | 0.124 |
| Excluded participants whose pancreatic cancer was not the first diagnosed cancer | 1.00 (reference) | 0.91 (0.68 to 1.22) | 1.06 (0.79 to 1.41) | 0.65 (0.46 to 0.93) | 0.017 |
| Repeated the analysis with energy-unadjusted total fried food consumption | 1.00 (reference) | 0.98 (0.74 to 1.29) | 1.01 (0.76 to 1.35) | 0.76 (0.55 to 1.06) | 0.080 |
| Repeated the analysis with sex-specific quartiles | 1.00 (reference) | 0.92 (0.70 to 1.19) | 0.83 (0.63 to 1.10) | 0.73 (0.53 to 1.00) | 0.051 |
| Further adjusted for Healthy Eating Index-2015 ^d^ | 1.00 (reference) | 0.89 (0.67 to 1.17) | 0.98 (0.74 to 1.31) | 0.70 (0.50 to 0.98) | 0.042 |
| Further adjusted for physical activity, and intakes of fruits, vegetable, red and processed meat, and coffee ^e^ | 1.00 (reference) | 0.87 (0.66 to 1.16) | 0.96 (0.72 to 1.28) | 0.67 (0.48 to 0.95) | 0.028 |
| **Deep-fried food consumption and pancreatic cancer** | | | | | |
| Excluded participants with extreme values of energy intake ^b^ | 1.00 (reference) | 0.82 (0.62 to 1.08) | 0.87 (0.66 to 1.15) | 0.63 (0.46 to 0.86) | 0.008 |
| Excluded participants with extreme deep-fried food consumption ^c^ | 1.00 (reference) | 0.76 (0.58 to 1.00) | 0.81 (0.61 to 1.06) | 0.58 (0.42 to 0.80) | 0.004 |
| Excluded cases occurred within the first two years of follow-up | 1.00 (reference) | 0.86 (0.64 to 1.15) | 0.90 (0.67 to 1.21) | 0.65 (0.46 to 0.92) | 0.021 |
| Excluded subjects whose pancreatic cancer was not the first diagnosed cancer | 1.00 (reference) | 0.83 (0.62 to 1.10) | 0.89 (0.67 to 1.18) | 0.59 (0.42 to 0.83) | 0.004 |
| Repeated the analysis with energy-unadjusted deep-fried food consumption | 1.00 (reference) | 0.92 (0.70 to 1.21) | 0.92 (0.70 to 1.22) | 0.68 (0.49 to 0.94) | 0.015 |
| Repeated the analysis with sex-specific quartiles | 1.00 (reference) | 0.87 (0.67 to 1.13) | 0.76 (0.58 to 1.00) | 0.70 (0.51 to 0.94) | 0.025 |
| Further adjusted for Healthy Eating Index-2015 ^d^ | 1.00 (reference) | 0.81 (0.61 to 1.06) | 0.86 (0.66 to 1.14) | 0.63 (0.46 to 0.87) | 0.010 |
| Further adjusted for physical activity, and intakes of fruits, vegetable, red and processed meat, and coffee ^e^ | 1.00 (reference) | 0.79 (0.60 to 1.04) | 0.85 (0.65 to 1.12) | 0.62 (0.45 to 0.85) | 0.008 |
| **Pan-fried food consumption and pancreatic cancer** | | | | | |
| Excluded participants with extreme values of energy intake ^b^ | 1.00 (reference) | 0.86 (0.64 to 1.15) | 1.07 (0.81 to 1.41) | 0.98 (0.72 to 1.31) | 0.849 |
| Excluded participants with extreme pan-fried food consumption ^c^ | 1.00 (reference) | 0.86 (0.64 to 1.15) | 1.05 (0.79 to 1.39) | 0.98 (0.72 to 1.34) | 0.782 |
| Excluded cases occurred within the first two years of follow-up | 1.00 (reference) | 0.88 (0.64 to 1.21) | 1.12 (0.83 to 1.51) | 1.04 (0.76 to 1.44) | 0.577 |
| Excluded subjects whose pancreatic cancer was not the first diagnosed cancer | 1.00 (reference) | 0.82 (0.60 to 1.11) | 1.02 (0.76 to 1.36) | 0.97 (0.71 to 1.32) | 0.762 |
| Repeated the analysis with energy-unadjusted pan-fried food consumption | 1.00 (reference) | 1.14 (0.85 to 1.51) | 1.14 (0.86 to 1.52) | 1.12 (0.82 to 1.52) | 0.719 |
| Repeated the analysis with sex-specific quartiles | 1.00 (reference) | 0.99 (0.75 to 1.31) | 1.04 (0.79 to 1.38) | 1.02 (0.76 to 1.37) | 0.874 |
| Further adjusted for Healthy Eating Index-2015 ^d^ | 1.00 (reference) | 0.87 (0.65 to 1.16) | 1.06 (0.80 to 1.41) | 0.99 (0.73 to 1.34) | 0.806 |
| Further adjusted for physical activity, and intakes of fruits, vegetable, red and processed meat, and coffee ^e^ | 1.00 (reference) | 0.85 (0.64 to 1.14) | 1.04 (0.78 to 1.37) | 0.95 (0.70 to 1.29) | 0.972 |

^a^ Values are hazard ratios (95% confidence intervals). Hazard ratios were adjusted for the following variables unless otherwise specified: age (years), sex (male, female), smoking status [current (>60 pack-years, 30–60 pack-years, <30 pack-years), former (>60 pack-years, 30–60 pack-years, <30 pack-years), never], alcohol consumption (g/day), body mass index (kg/m^2^), aspirin use (yes, no), history of diabetes (yes, no), family history of pancreatic cancer (yes, no), and energy intake from diet (kcal/day).

^b^ Extreme values of energy intake were defined as <800 or >4000 kcal/d for males and <500 or >3500 kcal/d for females.

^c^ Extreme consumption referred to top 2.5% or bottom 2.5% of indicated fried food consumption.

^d^ Healthy Eating Index-2015 was treated as a continuous variable in the multivariable Cox regression.

^e^ These covariates were treated as continuous variables in the multivariable Cox regression.

| **Supplemental Table 8.** Hazard ratios of the association between individual fried food consumption and the risk of pancreatic cancer ^a^ | | | | | |
| --- | --- | --- | --- | --- | --- |
| Individual fried food | Quartile 1 | Quartile 2 | Quartile 3 | Quartile 4 | *P*_trend_ |
| Deep-fried foods | | | | | |
| Deep-fried chicken | 1.00 (reference) | 0.93 (0.68 to 1.28) | 0.81 (0.56 to 1.19) | 0.94 (0.67 to 1.30) | 0.777 |
| Fried fish | 1.00 (reference) | 1.19 (0.91 to 1.56) | 0.96 (0.72 to 1.28) | 0.94 (0.70 to 1.25) | 0.308 |
| Fried potatoes | 1.00 (reference) | 0.92 (0.69 to 1.21) | 1.06 (0.81 to 1.39) | 0.80 (0.59 to 1.08) | 0.170 |
| Chips | 1.00 (reference) | 0.85 (0.64 to 1.12) | 0.88 (0.67 to 1.15) | 0.68 (0.50 to 0.90) | **0.015** |
| Pan-fried foods | | | | | |
| Pan-fried bacon | 1.00 (reference) | 1.03 (0.78 to 1.37) | 1.01 (0.76 to 1.34) | 0.97 (0.73 to 1.28) | 0.722 |
| Pan-fried chicken | 1.00 (reference) | 0.87 (0.63 to 1.19) | 0.66 (0.45 to 0.98) | 0.76 (0.54 to 1.06) | 0.501 |
| Pan-fried hamburger | 1.00 (reference) | 0.71 (0.51 to 0.98) | 0.81 (0.60 to 1.11) | 0.97 (0.74 to 1.27) | 0.344 |
| Pan-fried pork chops | 1.00 (reference) | 0.96 (0.69 to 1.32) | 0.86 (0.58 to 1.26) | 0.94 (0.67 to 1.33) | 0.898 |
| Pan-fried sausage | 1.00 (reference) | 1.00 (0.75 to 1.33) | 0.74 (0.55 to 1.00) | 0.96 (0.73 to 1.26) | 0.649 |
| Pan-fried steak | 1.00 (reference) | 0.65 (0.47 to 0.91) | 0.71 (0.49 to 1.03) | 0.88 (0.66 to 1.18) | 0.378 |

^a^ Hazard ratios were adjusted for age (years), sex (male, female), smoking status [current (>60 pack-years, 30–60 pack-years, <30 pack-years), former (>60 pack-years, 30–60 pack-years, <30 pack-years), never], alcohol consumption (g/day), body mass index (kg/m^2^), aspirin use (yes, no), history of diabetes (yes, no), family history of pancreatic cancer (yes, no), and energy intake from diet (kcal/day).

| **Supplemental Table 9.** Hazard ratios of the association between fried food consumption and the risk of pancreatic cancer with further adjustment for the indicated individual fried food ^a^ | | | | | |
| --- | --- | --- | --- | --- | --- |
| Individual fried food adjusted | Quartile 1 | Quartile 2 | Quartile 3 | Quartile 4 | *P*_trend_ |
| Total fried foods and pancreatic cancer | | | | | |
| Deep-fried chicken | 1.00 (reference) | 0.90 (0.68 to 1.18) | 1.01 (0.76 to 1.33) | 0.73 (0.52 to 1.02) | 0.080 |
| Fried fish | 1.00 (reference) | 0.89 (0.67 to 1.18) | 0.99 (0.75 to 1.30) | 0.69 (0.49 to 0.98) | 0.045 |
| Fried potatoes | 1.00 (reference) | 0.89 (0.67 to 1.18) | 0.99 (0.75 to 1.30) | 0.68 (0.47 to 0.98) | 0.050 |
| Chips | 1.00 (reference) | 0.92 (0.70 to 1.21) | 1.06 (0.80 to 1.41) | 0.82 (0.59 to 1.15) | **0.287** |
| Pan-fried bacon | 1.00 (reference) | 0.88 (0.66 to 1.16) | 0.95 (0.72 to 1.26) | 0.64 (0.46 to 0.90) | 0.012 |
| Pan-fried chicken | 1.00 (reference) | 0.90 (0.68 to 1.19) | 1.01 (0.76 to 1.33) | 0.74 (0.53 to 1.03) | 0.047 |
| Pan-fried hamburger | 1.00 (reference) | 0.89 (0.67 to 1.18) | 0.99 (0.75 to 1.30) | 0.69 (0.49 to 0.97) | 0.035 |
| Pan-fried pork chops | 1.00 (reference) | 0.89 (0.68 to 1.18) | 0.99 (0.75 to 1.31) | 0.70 (0.50 to 0.98) | 0.042 |
| Pan-fried sausage | 1.00 (reference) | 0.89 (0.68 to 1.18) | 0.99 (0.75 to 1.31) | 0.70 (0.50 to 0.98) | 0.043 |
| Pan-fried steak | 1.00 (reference) | 0.90 (0.68 to 1.18) | 1.00 (0.76 to 1.32) | 0.72 (0.51 to 1.00) | 0.058 |
| Deep-fried foods and pancreatic cancer | | | | | |
| Deep-fried chicken | 1.00 (reference) | 0.82 (0.62 to 1.07) | 0.88 (0.67 to 1.16) | 0.66 (0.47 to 0.91) | 0.021 |
| Fried fish | 1.00 (reference) | 0.81 (0.62 to 1.06) | 0.86 (0.65 to 1.13) | 0.60 (0.43 to 0.85) | 0.008 |
| Fried potatoes | 1.00 (reference) | 0.81 (0.62 to 1.06) | 0.86 (0.65 to 1.13) | 0.59 (0.41 to 0.84) | 0.008 |
| Chips | 1.00 (reference) | 0.83 (0.63 to 1.09) | 0.93 (0.71 to 1.23) | 0.74 (0.53 to 1.03) | **0.133** |

^a^ Hazard ratios were adjusted for age (years), sex (male, female), smoking status [current (>60 pack-years, 30–60 pack-years, <30 pack-years), former (>60 pack-years, 30–60 pack-years, <30 pack-years), never], alcohol consumption (g/day), body mass index (kg/m^2^), aspirin use (yes, no), history of diabetes (yes, no), family history of pancreatic cancer (yes, no), energy intake from diet (kcal/day), and the indicated individual fried food consumption (g/day).

| **Supplemental Table 10.** Hazard ratios of the association between fried food consumption and the risk of pancreatic cancer after omitting the indicated individual fried food in each turn ^a^ | | | | | |
| --- | --- | --- | --- | --- | --- |
| Individual fried food omitted | Quartile 1 | Quartile 2 | Quartile 3 | Quartile 4 | *P*_trend_ |
| Total fried foods and pancreatic cancer | | | | | |
| Deep-fried chicken | 1.00 (reference) | 0.90 (0.68 to 1.19) | 0.97 (0.73 to 1.28) | 0.74 (0.54 to 1.03) | 0.082 |
| Fried fish | 1.00 (reference) | 1.11 (0.84 to 1.47) | 1.09 (0.82 to 1.44) | 0.80 (0.58 to 1.11) | 0.073 |
| Fried potatoes | 1.00 (reference) | 1.01 (0.77 to 1.32) | 0.88 (0.66 to 1.16) | 0.74 (0.54 to 1.02) | 0.036 |
| Chips | 1.00 (reference) | 0.94 (0.71 to 1.25) | 1.08 (0.82 to 1.43) | 0.78 (0.56 to 1.08) | **0.122** |
| Pan-fried bacon | 1.00 (reference) | 0.96 (0.73 to 1.27) | 0.94 (0.71 to 1.24) | 0.70 (0.50 to 0.97) | 0.022 |
| Pan-fried chicken | 1.00 (reference) | 0.91 (0.68 to 1.20) | 1.05 (0.80 to 1.39) | 0.77 (0.56 to 1.07) | **0.135** |
| Pan-fried hamburger | 1.00 (reference) | 0.79 (0.60 to 1.04) | 0.90 (0.68 to 1.18) | 0.67 (0.48 to 0.92) | 0.033 |
| Pan-fried pork chops | 1.00 (reference) | 0.85 (0.64 to 1.12) | 0.97 (0.74 to 1.28) | 0.72 (0.52 to 1.00) | 0.073 |
| Pan-fried sausage | 1.00 (reference) | 0.95 (0.72 to 1.25) | 1.01 (0.77 to 1.33) | 0.68 (0.49 to 0.95) | 0.016 |
| Pan-fried steak | 1.00 (reference) | 0.86 (0.65 to 1.15) | 1.01 (0.77 to 1.34) | 0.77 (0.56 to 1.07) | **0.161** |
| Deep-fried foods and pancreatic cancer | | | | | |
| Deep-fried chicken | 1.00 (reference) | 0.87 (0.67 to 1.14) | 0.86 (0.65 to 1.13) | 0.66 (0.49 to 0.91) | 0.014 |
| Fried fish | 1.00 (reference) | 1.14 (0.87 to 1.49) | 0.98 (0.74 to 1.30) | 0.79 (0.58 to 1.09) | 0.044 |
| Fried potatoes | 1.00 (reference) | 1.05 (0.80 to 1.36) | 0.91 (0.69 to 1.19) | 0.74 (0.54 to 0.99) | 0.020 |
| Chips | 1.00 (reference) | 0.99 (0.75 to 1.31) | 0.96 (0.72 to 1.27) | 0.83 (0.61 to 1.13) | **0.180** |

^a^ Hazard ratios were adjusted for age (years), sex (male, female), smoking status [current (>60 pack-years, 30–60 pack-years, <30 pack-years),

former (>60 pack-years, 30–60 pack-years, <30 pack-years), never], alcohol consumption (g/day), body mass index (kg/m^2^), aspirin use (yes, no), history of diabetes (yes, no), family history of pancreatic cancer (yes, no), and energy intake from diet (kcal/day.

**STROBE Statement—Checklist of items that should be included in reports of *cohort studies***

|  | Item No | Recommendation | Page No |
| --- | --- | --- | --- |
| **Title and abstract** | 1 | (*a*) Indicate the study’s design with a commonly used term in the title or the abstract | 1 |
|  |  | (*b*) Provide in the abstract an informative and balanced summary of what was done and what was found | 1 |
| Introduction | | | |
| Background/rationale | 2 | Explain the scientific background and rationale for the investigation being reported | 2 |
| Objectives | 3 | State specific objectives, including any prespecified hypotheses | 2 |
| Methods | | | |
| Study design | 4 | Present key elements of study design early in the paper | S1 |
| Setting | 5 | Describe the setting, locations, and relevant dates, including periods of recruitment, exposure, follow-up, and data collection | S1 |
| Participants | 6 | (*a*) Give the eligibility criteria, and the sources and methods of selection of participants. Describe methods of follow-up | S |
|  |  | (*b*) For matched studies, give matching criteria and number of exposed and unexposed |  |
| Variables | 7 | Clearly define all outcomes, exposures, predictors, potential confounders, and effect modifiers. Give diagnostic criteria, if applicable | S1 |
| Data sources/ measurement | 8* | For each variable of interest, give sources of data and details of methods of assessment (measurement). Describe comparability of assessment methods if there is more than one group | S1 |
| Bias | 9 | Describe any efforts to address potential sources of bias | S2-3 |
| Study size | 10 | Explain how the study size was arrived at | (NA) |
| Quantitative variables | 11 | Explain how quantitative variables were handled in the analyses. If applicable, describe which groupings were chosen and why | S2-3 |
| Statistical methods | 12 | (*a*) Describe all statistical methods, including those used to control for confounding | S2-3 |
|  |  | (*b*) Describe any methods used to examine subgroups and interactions |  |
|  |  | (*c*) Explain how missing data were addressed |  |
|  |  | (*d*) If applicable, explain how loss to follow-up was addressed |  |
|  |  | (*e*) Describe any sensitivity analyses |  |
| Results | | |  |
| Participants | 13* | (a) Report numbers of individuals at each stage of study—eg numbers potentially eligible, examined for eligibility, confirmed eligible, included in the study, completing follow-up, and analysed | 2, 3 |
|  |  | (b) Give reasons for non-participation at each stage |  |
|  |  | (c) Consider use of a flow diagram |  |
| Descriptive data | 14* | (a) Give characteristics of study participants (eg demographic, clinical, social) and information on exposures and potential confounders | 3 |
|  |  | (b) Indicate number of participants with missing data for each variable of interest |  |
|  |  | (c) Summarise follow-up time (eg, average and total amount) |  |
| Outcome data | 15* | Report numbers of outcome events or summary measures over time | 3 |

| Main results | 16 | (*a*) Give unadjusted estimates and, if applicable, confounder-adjusted estimates and their precision (eg, 95% confidence interval). Make clear which confounders were adjusted for and why they were included | 3 |
| --- | --- | --- | --- |
|  |  | (*b*) Report category boundaries when continuous variables were categorized |  |
|  |  | (*c*) If relevant, consider translating estimates of relative risk into absolute risk for a meaningful time period |  |
| Other analyses | 17 | Report other analyses done—eg analyses of subgroups and interactions, and sensitivity analyses | 3 |
| Discussion | | | |
| Key results | 18 | Summarise key results with reference to study objectives | 4 |
| Limitations | 19 | Discuss limitations of the study, taking into account sources of potential bias or imprecision. Discuss both direction and magnitude of any potential bias | 4, 5 |
| Interpretation | 20 | Give a cautious overall interpretation of results considering objectives, limitations, multiplicity of analyses, results from similar studies, and other relevant evidence | 6 |
| Generalisability | 21 | Discuss the generalisability (external validity) of the study results | 6 |
| Other information | | | |
| Funding | 22 | Give the source of funding and the role of the funders for the present study and, if applicable, for the original study on which the present article is based | 6 |

*Give information separately for exposed and unexposed groups.

**Note:** An Explanation and Elaboration article discusses each checklist item and gives methodological background and published examples of transparent reporting. The STROBE checklist is best used in conjunction with this article (freely available on the Web sites of PLoS Medicine at http://www.plosmedicine.org/, Annals of Internal Medicine at http://www.annals.org/, and Epidemiology at http://www.epidem.com/). Information on the STROBE Initiative is available at http://www.strobe-statement.org.
